# A Contribution to the Study of the Ætiology, Symptomatology and Treatment of Endometritis

**Published:** 1895-10

**Authors:** George Tucker Harrison

**Affiliations:** New York


### Southern Medical Record.

A MONTHLY JOURNAL OF MEDICINE AND SURGERY.

VOL. XXV. ATLANTA, GA., OCTOBER, 1895. No. 10.

### Original Articles.

### A CONTRIBUTION TO THE STUDY OF THE ÆTIOLOGY, SYMPTOMATOLOGY AND TREATMENT OF ENDOMETRITIS.

BY GEORGE TUCKER HARRISON, M. A., M. D., NEW YORK.

Endometritis is the most frequent gynæcological disease. Its significance ranges from the slight annoyances of a simple uterine catarrh, to the dread train of symptoms pertaining to a septic endometritis which has become the point of departure to a general infection. Even in its simple forms, while not jeopardizing life, it may cause such symptoms as to make existence far from enjoyable. Although it cannot be said that it is a part of the gynæcological field which has been neglected, there is yet fruit to be gathered. It is of paramount importance, at the outset, to classify the various forms of endometritis and, in order to do this, it seems best to have regard to the ætiological factors which modern bacteriological investigation has elicited. With Von Winckel we may divide all the varieties of endometritis into two great classes:

1. Simple forms of endometritis, *i. e.*, those in which the bacterial origin has not been demonstrated.—(*a*) endometritis due to disturbances in the circulation; (*b*) endometritis from toxic agents or from general infection; (*c*) endometritis *post abortum* or *deciduale*. (*d*) *endometritis exfoliativa*.

2. Purulent forms of endometritis of bacteritic origin. Here belong (*e*) gonorrhœal endometritis; (*f*) tubercular endometritis; (*g*) endometritis due to streptococci; (*h*) saprophytic endometritis; (*i*) diphtheritic endometritis; (*j*) syphilitic endometritis; (*k*) endometritis from blastomycetes; (*l*) endometritis due to the presence of protozoa.

It is obvious that such a classification should only be regarded as provisional, and, with advancing knowledge, it may become necessary to transfer certain members of the first group to the second. It will be noticed that most of the forms grouped under the first class belong to chronic endometritis. The group (*a*) comprises the various forms of uterine catarrh. It may be observed in the young girl, as often associated with chronic constipation and, ætiologically, may be brought into relation with imprudences of dress, neglect of proper precautions during menstruation or imperfect development of the uterus. The most frequent causes, however, are lesions occurring during childbirth, want of proper treatment in the puerperium, retrodeviations, prolapsus and myomata. I shall not dwell on the second group of causes, merely recalling to your memory that general infectious diseases like *cholera*, *small-pox*, *scarlet fever*, *measles*, *typhoid fever*, *etc.*, may have, as a consequence, uterine catarrh. As a result of cholera, according to Slamonesky, entravasation may take place, pervading the entire thickness of the mucous membrane leading to its detachment, so that it projects free into the cavity of the uterus. Here belongs that form of endometritis which Brennecke first pointed out, and which Czempin's observation tended to confirm, due to oophoritis and other affections of the *ovaries* and *annexa*. As this author's views may not be familiar to all of you I quote a passage from his article which explains his views: "*Chronic hyperplastic endometritis*," he remarks, "is to be regarded as a pure hyperplasia of the mucous membrane of the uterus. It appears in consequence of a chronic hyperæmia of the uterine mucous membrane and this is maintained, in a reflex way, by abnormal irritations that affect the nervous reflex apparatus, liberating the menstrual congestions, in ovaries which are physiologically or pathologically changed and thence performing their functions abnormally." It is certainly premature to endeavor to make an exact differential diagnosis between the pure uterine endometritis and the ovarian form as Brennecke attempts to do. According to the beautiful and exact studies of K. Ruge these forms of endometritis, histologically, may be separated into a *glandular*, an *interstitial*, and a mixed form. The *endometritis fungosa* or hyperplastic endometritis of Olshausen may be a purely *interstitial* form or it may be a *hypertrophic glandular*, or, finally, it may be a *hyperplastic glandular endometritis*. *Endometritis post abortum* is considered in a separate class corresponding to its importance. The inflammatory affections so frequently following upon abortions are due to the fact that the *mucous* membrane, instead of a return to a normal state, passes into a condition of inflammatory *proliferation* under the influence of the exciting causes potent in these circumstances. Here especially we find the interstitial form, but at other times, especially after abortions in the early months of pregnancy, we meet forms of *endometritis glandularis hyperplastica*. In other cases the inflammatory phenomena may be referred to defective involution of the decidua. Instead of the cells of the decidua diminishing uniformly in the entire mucous membrane, the processes of involution occur with different degrees of rapidity in different places; larger or smaller islets of decidua maintain themselves surrounded by a round-celled proliferation of the mucous membrane. Küstner* has proposed the name of *deciduoma* to designate these peculiar ætiological relations. The same ætiological view serves to explain the origin of those small flat, polypus-like proliferations so often found in the angles corresponding to the tubes. The *endometritis exfoliativa* formerly called membranous endometritis, exhibits, histologically two forms, the ordinary interstitial form and that in which the intercellular tissue is more largely involved than the stroma cells. The framework is thickened, recalling often the tough elastic bands of fibres of other tissues. The cells of the stroma, which do not appear especially increased, are forced apart by the exudation and now and then show changes, their nuclei enlarging and appearing plainly in the cellular body, so that it might be imagined that we had the preliminary stage in the formation of actual decidua cells. At times the stroma is pervaded with red blood corpuscles.

*Grundzüge der Gynækologie, p. 219.

Let us now consider the second great class of cases comprehended under the term endometritis—those of micro-parasitic origin. The three varieties which especially interest us from their importance and frequency, are the *gonorrhœal*, the *septic* and the *saprophytic*, from their frequency and pathological significance. *Tubercular endometritis* is, according to my observation, quite rare, the seat by preference of tuberculosis, when it attacks the genital organs in woman, being the tubes. *Gonorrhœal endometritis* is, unfortunately, of very frequent occurrence and is evoked by an invasion of the gonococci of Neiser. The inflammation produced in the uterine mucous membranes from this cause, according to Wertheim, may be called "*interstitial endometritis, with purulent catarrh.*" In a number of cases, a glandular endometritis may be developed. The inflammation may also invade the muscular tissue, producing an increase in the volume of the uterus and heightening its sensibility. The microbe, as is well-known, propagates itself by contiguity with the tubes and to the ovaries. Wertheim declares that his researches, in cases of recent gonorrhœa, have shown him that the internal os is not at all, as has been generally asserted, an obstacle to the invasion of the uterine cavity by the gonorrhœal infection. The *endometritis* due to *streptococci* is usually of puerperal origin—so too, with the *saprophytic* or *putrid endometritis*. The subject of puerperal endometritis has been studied by Bumm with fruitful results. As he insists, we must discriminate between a *putrid* and a *septic endometritis*. The latter is to be subdivided into (*a*) localized septic endometritis and (*b*) septic endometritis, followed by a general infection. In the putrid (saprophytic) endometritis, the underlying uterine wall very quickly and certainly forms a layer of granulation tissue, which effects the detachment of the diseased endometrium. So, too, in the localized septic endometritis, on the zone of bacteria-proliferation, follows a zone of reaction, the round-celled infiltration. The bacteria do not penetrate into the developed granulation tissues. The process is arrested at the uterine wall. It manifestly performs the function of a limiting wall to the pathogenic streptococci, as it does to the putrefactive germs. It is quite otherwise in the case of septic endometritis with general infection. Here, the emigration of leucocytes is wanting. The streptococci, and staphylococci in addition to saprophytic bacilli penetrate into the muscular wall and may even pass through it to the peritoneum, and thus evoke a fatal peritonitis. There are two and only two avenues by which the infective germs are carried farther, so as to produce a general infection—by the lymphatic and by the blood channels. Bumm is inclined to believe that the streptococci peritonitis, as a rule, originates by direct penetration of the germ through the uterine wall and only exceptionally by the roundabout way of the Fallopian tubes. Only where the pathologically changed oviduct is connected with the uterine cavity by a wide opening, do the germs take this route. In a septic infection of the endometrium, the physiological stenosis of the genital canal, at the *ostium uterinum tubæ*, operates as a well-closing valve to prevent the penetration of the germs. The symptoms of acute endometritis can hardly be depicted so as to give a clearly defined clinical picture, the accompanying general phenomena taking up the foreground. Endometritis may be diagnosticated in cases of general infection, when the uterus is tender on pressure, when its secretion is increased, and when there are disturbances referable to the neighboring organs. After gonorrhœal infection, the use of unclean sounds, or rough attempts at dilatation without strict regard to the principles of asepsis, the symptoms are better defined. In such circumstances, the uterus is found to be swollen and tender on pressure, the portio and cervix reddened, the secretion increased and purulent in character. The most important symptom of chronic endometritis is hemorrhage, either *menorrhagia* or *metrorrhagia*. The usual time of menstruation is anticipated and the duration is abnormally long. In other cases, the time of recurrence is postponed; amenorrhœa exists for some time, to be followed by profuse menstruation. To this type, belongs the ovarian hyperplastic endometritis of Brennecke. In the time between the periods, there is a more or less leucorrhœal discharge, which may be watery or purulent. The discharge varies greatly in quantity, being sometimes very slight, and in others excessively profuse, especially in gonorrhœal inflammation. The patients complain of a sensation of weight and pressure in the pelvis, of pain referable to the sacral region, sensations which attain their maximum intensity just before the menstrual period and are relieved usually with the discharge of blood. Occasionally, however, the annoying symptoms continue during the whole period. In some cases, pain is only felt in the middle of the time between two periods, for which Schroeder proposed the term "middle pain." Sensitiveness of the uterine mucous membranes to contact with the sound is characteristic of endometritis. Pain begins as soon as the sound passes the internal os and may be evoked by its contact with any part of the mucosa. Sterility is a frequent consequence of endometritis, but even in cases in which conception takes place, there is always danger of an *abortion*, nay, at times, of *placenta prævia*. Of symptoms affecting remote organs, mention should be made of nervous dyspepsia, of persistent headache, and when the disease is of long standing, that distressing train of symptoms comprehended under the term neurasthenia. My observations agree with those of Fehling, when he asserts that these latter are found more frequently in the forms characterized by scanty purulent secretion, than in cases of copious discharge, which finds no obstacle to its escape. It is interesting to note that gonorrhœal endometritis is by no means infrequently accompanied by *stenosis* of the external os.

In correspondence with its supreme importance, the treatment of puerperal endometritis must be first considered. It seems scarcely necessary at this time to insist upon the absolute necessity of seeing to it that, in the interest of prophylaxis, the hands of the obstetrician who attends a parturient woman should be thoroughly clean, in the surgical sense, and that his instruments should be sterilized before using, and that, moreover, the external genitalia should be carefully cleaned and disinfected before any examination. I still hold to the views I advocated in a paper read before you ten years ago, in which I advocated the importance of *subjective*, as opposed to *objective antisepsis*. As soon as symptoms indicative of septic endometritis arise, the uterine cavity should be cleansed of all foreign bodies, such as shreds of ovular membranes, blood-coagula, remains of placenta, etc., decidual pieces, by a copious intra-uterine irrigation. This may be followed by a second within six or eight hours. Repeated irrigations are unnecessary and useless. We should use a 3 to 4 per cent. warm solution of Calvert's carbolic acid or a 1 to 1½ per cent. solution of lysol. Corrosive sublimate, I do not use, on account of the danger of toxic effects. If no improvement follows the intra-uterine irrigation, the fever remaining high, it must be assumed that the infective germs have penetrated into the deeper tissues. Under the circumstances, the cautious use of the curette may yet bring relief. I am aware that, here, I am treading upon dangerous ground. Some of the most accomplished gynæcologists deprecate the use of the curette in puerperal cases, when birth has occurred at full term, in the early days of the puerperium, grounding their objections on the extent of surface to be operated upon, the facility with which the soft organs may be wounded or perforated, and, finally, upon the danger of carrying the infective germs into the vulnerable tissues and thus promoting the spread of the infection. I saw not long since, a case of *pyæmia* produced by a colleague who curetted a puerperal uterus four or five days after childbirth. I believe it should be employed in the circumstances just mentioned, only when the intra-uterine irrigation has failed us. Of course, there can be no question of the indication for *curettage* in cases of retained parts of the placenta or membranes in the later part of the puerperium and *post abortum*. After the operation, the uterine cavity should be irrigated with a lysol or carbolic acid solution, or sterilized solution of salt, as Pryor prefers. Fehling swabs out the uterus with a 50 per cent alcoholic solution of carbolic acid. The uterine cavity should be firmly packed with iodoform gauze. I would here insist upon it that recourse should not be had to the curette as a mere matter of routine, with out due consideration of the aim and object of its use. The beautiful studies of Bumm have shown us that in cases of saprophytic and localized septic endometritis, nature has, as it were, already anticipated us and by the formation of a zone of reaction, the round-celled infiltration, made provision against a deeper penetration of the microbes and at the same time effected a mechanism by which the necrotic masses are detached to make their escape with the lochial discharge. It is easy to see that the rough and incautious use of the curette might injuriously affect the delicate adjustment. In cases of abortion, when the ovum shows signs of decomposition, or retained portions of the ovum are undergoing putrefactive decomposition, curettage is indicated, and imperatively so when inflammatory phenomena in the uterus and vicinity are observed, associated with high fever and septic conditions. The removal of the decomposing masses, the thorough disinfection of the uterine cavity, followed by packing the uterine cavity, change the clinical picture in a short time in a most gratifying way. The fact should not be overlooked that an intra-uterine douche is not a matter of indifference, especially a uterus from which a fœtus has been expelled, belonging to the second half of fœtal life. Several times it has been my lot to witness the following phenomena after an intra-uterine irrigation of the puerperal uterus with a solution of carbolic acid: The patient was suddenly attacked with dyspnœa, the pulse became small and frequent, then convulsions came on, with a total loss of consciousness. I at once stopped the flow of the solution irrigating the uterine cavity and in a comparatively short time, the patient recovered. The phenomena were evidently elicited by the entrance of carbolic acid into the blood channels. In reference to the treatment of gonorrhœal endometritis, I cannot do better than call your attention to the views and experience of my friend, Dr. Wm. R. Pryor, as he has worked in this direction with assiduity for some time. It will be seen that he has opened up to us a field of splendid therapeutical endeavor. With insignificant exceptions, I concur heartily in the views he advocates. He has been kind enough to give me the following condensed statement of his views:

*Gonorrhœal Endometritis.* Uncomplicated by pelvic involvement.

If necessary, deep cervical incisions to admit of dilatation of at least one-half to three-fourths inch. Less will not do.

Dilatation.

Curettage thorough.

Irrigation with salt solution through as large a Fritsch-Bozeman catheter as will pass. This is necessary in order to remove all debris.

Pack uterus with special iodoform gauze by means of heavy applicator, not using a cervical speculum. A nulliparous uterus will hold three feet by one or two inches of gauze. Vagina also packed. Cervical incision not sewed up.

Fresh dressing in from five to ten days. Uterine packing not renewed.

Vagina kept packed until new endometrium is formed, usually in four weeks. No intercourse until two periods after operation have passed.

Number of cases operated upon very large. Cannot give figures. Routine procedures in my clinics. Have absolutely never seen a complication follow. I lay stress upon following:

1. Preliminary cleansing of part by brushes and lysol, 2%.
2. Wide cervical dilatation.
3. Removal of all debris.
4. Irrigating with non-irritating solution.
5. Firm, full packing of uterus.

*Gonorrhœal Endometritis.* Acute, with complications.

A. Seventeen curettages, with subsequent cœliotomy in three cases. These latter were seen after infection had lasted over one week.

All cases typical, vomiting, temperature, tympanitis, profuse discharge, uterus fixed, one or both tubes easily feel enlarged under narcosis. In fourteen cases every sign of local disease disappeared. In three cases the local condition benefited.

Technique same as in uncomplicated endometritis.

B. In six cases with bilateral pyosalpinx, curettage as before. Cul-de-sac opened; pus tubes ripped open; pelvic Mikulicz of iodoform gauze.

All young women under twenty-seven years, did the partial operation to avoid genital atrophy and artificial menopause.

First operation seven months old.

*Results:* Uterus fixed in pelvis more or less; menstruation in every respect normal; coitus painless; endometritis *none.*

In every case I look for a relapse or rather a re-infection, because they will return to infected husbands or lovers.

If unilateral pyosalpinx is present, I remove it per vaginam.

In four cases of gonorrhœal endometritis with acute salpingitis, curettage, cul-de-sac opened, adhesions broken up, pelvic Mikulicz. Immediate results perfect.

*Gonorrhœal Endometritis.* Chronic, with complications.

A. Vaginal hysterectomy in bilateral lesions always as the preferred method.

*Observations:* Sixteen suprapubic hysterectomies, one death from appendicitis. Twenty-one vaginal hysterectomies without a death.

In all cases I used the pelvic Mikulicz.

B. Where extraneous circumstances modify this preferable operation, as youth, refusal to submit to it, etc., I curette and open the cul-de-sac, breaks up all adhesions, pack pelvis with gauze. Thus I get the drainage at both ends of tubes and cut off the original source of infection at the same time.

*Observations:* As near as I can recall without going over records, ten or twelve. Immediate result excellent, patient symptomatically cured. Uterus remains more or less fixed. Ultimate results, I cannot foretell.

C. Curettage alone in chronic cases which have had repeated claps.

*Observations:* Eighty-seven; eleven subsequent cœliotomies or vaginal sections, *not* because curettage caused complications to increase, but because gross changes not removed by the curettage.

In two cases of pyosalpinx the tubes drained into the uterus, with a resulting re-infection. This result is so rare as to be ignored. In all my life I have seen but two pus tubes so evacuate. Gill Wylie had one of these.

SUMMARY.—A case of *uncomplicated* purulent endometritis, acute or chronic—curettage.

Complicated by tubal disease—curettage, cul-de-sac incision, breaking up adhesions, opening widely all pus foci, pelvic Mikulicz.

This every general practitioner can do, after once seeing it done.

The one radical, sure, permanently successful method for bilateral suppurative disease is ablation, preferably vaginal; ablation of uterus and tubes in bilateral disease; removal of one tube where that alone is involved.

I cannot too strongly recommend the cul-de-sac incision as a supplementary procedure to the curettage in complicated cases. I have done it but once, in a strictly post-partum case, where three other men had curetted alone, and with perfect result.

Anything to save the uterus, menstrual functions, and prevent genital atrophy and artificial menopause.

PALLIATIVE MEASURES.—*Curettage alone* in chronic complicated endometritis, and in some cases of acute complicated.

*Curettage and cul-de-sac incision* in same class of cases.

RADICALLY CURING.—*Curettage* in all cases of uncomplicated endometritis and in 80 per cent acute complicated.

*Curettage and cul-de-sac* method always successful in acute complicated, so far as tried. Same with chronic complicated. Too soon to know ultimate results.

*Hysterectomy* in all cases successful.

| | GONORRHŒAL ENDOMETRITIS. | ACUTE COMPLICATED. | CHRONIC COMPLICATED. |
|---|---|---|---|
| *Curettage alone*...... | Great number of both acute and chronic cases. Results uniformly satisfactory. Have always succeeded in preventing complications. | 17 cases. 14 c red. 3 subsequent cœliotomies. | 87 cases. 11 subsequent cœliotomies. Rest cured of all symptoms. |
| Curettage and cul-de-sac opened............. | | 6 cases with acute pyosalpinx. 4 cases with acute suppurative; salpingitis similar to the above 17. All cured of symptoms *so far*. | 12 cases. Results I believe to be permanent I exclude here pyosalpinx, and include occluded tubes, hydrosalpinx, adherent tubes and ovaries. |
| Hysterectomy ........... | | 16 suprapubic operations—1 death. 21 vaginal operations—no deaths. | |

In dealing with cases of chronic endometritis, the chief point to know is which cases demand local measures and which are relieved by general treatment. Chlorotic and anæmic young girls, for example, who suffer from uterine catarrh, should not be subjected to local treatment, as a rule, but placed upon such a course of general treatment as will improve the quality of the blood and strengthen the body. The best means at our command, in the way of local therapeutics, are dilatation of the cervical canal, curettage, the uterine tampon and cauterization in a certain class of cases. Dilatation by laminaria tents, I hardly ever employ, although it can now be made an aseptic measure for the reason that with a large curette, after rapid dilatation it is possible to feel any projection and inequality of the mucous membrane. The class of cases belonging to this category, in which the curette is indicated, comprises all forms of *endometritis fungosa* and *endometritis exfoliativa*. In catarrhal endometritis, less is gained by its use, as a rule. The indications in gonorrhœal endometritis we have already studied. There is a difference of opinion as to the best instrument for dilatation. For my part, I prefer graduated steel sounds. The various modifications of Ellenger's dilator, consisting of two blades, have their zealous advocates. It is not so much the instrument as it is the operator that is of importance. The chief point is that it should be an absolutely aseptic procedure. The patient may be either in the lithotomy or Sim's position; I use both. Before operation the vulva should be thoroughly cleansed and all instruments sterilized. The vagina and portio are thoroughly cleansed with a corrosive chloride or lysol solution by rubbing them with borated cotton dipped in the disinfecting solution. The cervical canal may be disinfected by a piece of cotton wrapped round an applicator and dipped in a five per cent solution of carbolic acid. The *portio* is seized with a bullet forceps and the cervix thoroughly dilated, passing in one sound in succession. Then follows the curettage. We can easily ascertain when the mucosa is scraped off by the sensation imparted to the hand by the curette and by the peculiar grating sound conveyed to the ear. After the completion of the curettage, the uterus should be irrigated with a disinfecting solution by means of a Fritsch-Bozeman catheter. Then follows the introduction of a tampon of iodoform gauze into the uterus up to the fundus, using a strip of gauze two to three centimeters in width. In order to be sure that the uterus is thoroughly clean, the piece first introduced may be taken out and a second introduced. The uterine cavity must be firmly packed. A great many entirely mistake the function of the iodoform gauze and thrust a strip of gauze into the uterine canal for the purpose, as they say, of drainage only. This is a great error. Then follows a vaginal tampon. When firmly packed, the gauze comes in contact with the entire inner surface of the uterus. It absorbs the secretions, disinfects them and maintains them, in an aseptic condition, and, at the same time, prevents all hemorrhage. The tampon should be retained *in situ* from five to seven or ten days. After the removal of the uterine tampon and there is still a discharge, if the cavity of the uterus is large, tincture of iodine should be applied after irrigation. The iodine treatment may be repeated after five or six days. In this way the uterus becomes smaller, and the new mucosa, undergoing regeneration in a smaller space, will not become as thick as formerly. Lastly, it must be borne in mind that there are certain types of endometritis, which, although not of a malignant type, bid defiance to our therapeutic endeavors, and in these cases, the question of total extirpation may well be brought to discussion.

---

LOFTY STATUE VERSUS LONG LIFE.—A writer in the *National Popular Review* has been investigating the age period at death of those abnormally tall individuals who are classed as "giants," and believes he has found that all who measure over six and one-half feet have been short in their life's span. He is of the opinion that there is sure to be some failing link in the physiologic chain of these abnormal beings; and this, too, independently of any of those moral inadequacies which too often exist and help to precipitate an early downfall or breakdown. He further says:

"As a rule, giants are not long-lived. They have too many gauntlets to run. Being giants—that being anything over six feet six—they naturally drift into the show business and are thenceforth incarcerated in vans, close rooms and in the dingy and effluvia-laden air of the exhibition room. Their not over-resisting lungs here inhale the combined effluvia and aroma that arises from the lungs, skin and not overclean or over-well-aired clothes of their many admirers, all of which is not conducive to either health or to long life. It would seem reasonable to believe that a giant—be he seven or ten feet tall—who is well formed, and who has every organ in a just proportion to his bulk, should live as long as a small man or as long as his heredity might otherwise permit. Reasoning theoretically, this would seem probable, but when we come to analyze the subject and compare the actual facts, we find that something or other always goes wrong, and, owing to many an "if," we find that our giant dies early, as a rule. Some one organ goes wrong, and the great machine comes to a stop, or some organ does not keep pace with the rest of the increase in bulk, and he goes halting and squeaky, or either an overwork or an underwork here or there, and a physiologic inadequacy of some sort is the result, with a general deterioration of the whole structure and a finally premature death."—*The Journal.*

